# Ecosystem C:N:P Stoichiometry and Carbon Stocks Along a Chronosequence of *Malus pumila* Orchards in North China

**DOI:** 10.3390/plants15162502

**Published:** 2026-08-19

**Authors:** Haizhou You, Xiaoya Yu, Tao Zhang, Yanjie Qin, Huitao Shen

**Affiliations:** 1 Hebei Academy of Forestry and Grassland Science, Shijiazhuang 050061, China; youhaizhou1204@163.com; 2 School of Tourism and Resource Environment, Qiannan Normal University for Nationalities, Duyun 558000, China; ynyxy800305@163.com; 3 Hebei Technology Innovation Center for Geographic Information Application, Institute of Geographical Sciences, Hebei Academy of Sciences, Shijiazhuang 050021, China; 4 Station of Agro-Pastoral Ecotone in Bashang Hebei (Grassland), Ecological Quality Comprehensive Monitoring Station, Ministry of Ecology and Environment of People’s Republic of China, Chengde 068462, China

**Keywords:** ecological stoichiometry, carbon stocks, stand age, *Malus pumila* orchard, plant–soil system, Yan Mountain

## Abstract

Understanding the dynamics of carbon (C), nitrogen (N), and phosphorus (P) stoichiometry and C stocks along a stand development chronosequence has been extensively studied in forest ecosystems. However, despite the global economic and ecological importance of apple orchards, such knowledge remains limited for these intensively managed perennial agroecosystems. We examined C, N, and P concentrations and stoichiometric ratios in tree tissues (root, stem, branch, foliage) and soils (0–100 cm depth), as well as ecosystem C stocks, across a chronosequence of 4, 8, 12, and 16 yr old *Malus pumila* orchards in the eastern Yan Mountains, Hebei Province, North China. The results showed that C concentrations exhibited no consistent age-dependent trend in tree tissues. In contrast, N and P concentrations in all tree tissues decreased significantly with stand age, while their C:N and C:P ratios increased. The leaf N:P ratios suggested progressive P limitation as orchards aged. In soil, C, N, and P concentrations first decreased and then increased along the chronosequence, with the highest values observed in the 16 yr stands. This U-shaped trajectory reflected the dynamic interplay between stand development and anthropogenic management. Intercropping and intensive fertilization in the 4 yr orchards initially elevated soil nutrient levels, while the cessation of intercropping and nutrient removal via fruit harvesting in the 8 yr stands led to a decline. Thereafter, accumulation of litter decomposition and root turnover, combined with continued organic matter inputs, progressively replenished soil nutrient pools in the 12 and 16 yr stands. The total ecosystem C stocks ranged from 70.80 to 136.13 Mg ha^−1^, initially declining from 4 to 8 years and then increasing at 12 and 16 years, with soil contributing 84.7–99.7% of the total. Plant and soil nutrient concentrations showed predominantly negative correlations, indicating weak coupling between tree and soil nutrient pools. Our findings demonstrated that stand age profoundly influenced C:N:P stoichiometry and C stocks in apple orchard ecosystems and that prolonged orchard development enhanced both tree biomass C and soil C stocks. These results provide a scientific basis for nutrient optimization and sustainable management of apple orchards in temperate regions.

## 1. Introduction

Global climate change and the intensification of the greenhouse effect are currently the most severe challenges faced by nations around the world [1,2]. Enhancing the carbon sink function of terrestrial ecosystems to mitigate the continuous rise in atmospheric CO_2_ concentrations has become a consensus in the international community [3]. Forests cover approximately 41 million km^2^, account for about 30% of the global land surface, and their vegetation and soils store 60% of the carbon in terrestrial ecosystems, playing a vital role in maintaining the global carbon balance [4]. Increasing carbon stocks by expanding the area of planted forests can effectively improve and sustain regional environments and the global carbon balance, thereby mitigating the process of global climate change [5,6].

Ecological stoichiometry, which focuses on the balance and interactions of multiple chemical elements—particularly carbon (C), nitrogen (N), and phosphorus (P)—in ecological processes, has emerged as a powerful framework for understanding nutrient cycling, energy flow, and the functional status of terrestrial ecosystems [7,8]. Previous studies have explored the dynamics of C, N, and P stoichiometry along chronosequences in various forest types, revealing that stand age significantly influences nutrient concentrations and ratios in plant–soil systems. Regarding vegetation, Zhang et al. [9] found that the leaf N:P ratio of *Robinia pseudoacacia* plantations increased significantly with stand age, while Liu et al. [10] reported that the leaf N:P ratio of Masson’s pine (*Pinus massoniana*) first decreased and then increased. In contrast, the leaf N:P ratio of Chinese fir (*Cunninghamia lanceolata*) remained relatively stable from young to overmature stands [11]. As for soil C:N:P stoichiometry with increasing stand age, studies have shown either increasing [12,13], decreasing [14], first decreasing and then increasing [15], first rising and then reducing [16], or no change [17,18] trends. Thus, during the development of plantation ecosystems, the C:N:P stoichiometric characteristics of plants and soils vary considerably depending on climatic conditions, species composition, soil type, and management practices [19,20]. This high variability underscores the need for ecological stoichiometry studies under specific regional and vegetation conditions [21,22].

Apple is recognized as one of the four major fruit trees and the second-most consumed fruit worldwide due to its extensive cultivation, economic significance, and growing demand in health-conscious diets [23]. As perennial agroecosystems, apple orchards are subject to intensive anthropogenic management, including tillage, fertilization, intercropping, and fruit harvesting [24,25]. These practices collectively modulate soil physicochemical properties, nutrient availability, and plant–soil nutrient coupling [26,27]. Tillage alters soil structure and organic matter decomposition, affecting soil C, N, and P stoichiometry [28]. Fertilization, while enhancing soil fertility and carbon sequestration, also modifies soil C:N:P stoichiometry and may induce acidification and nutrient imbalances [29,30]. Intercropping in young orchards enhances organic carbon sequestration, nitrogen fixation, and phosphorus solubilization while improving microbial biomass and enzyme activities [25], with studies reporting up to 40% increases in surface soil organic matter [27]. Fruit harvesting removes substantial N, P, and K, profoundly influencing long-term nutrient budgets and stoichiometric equilibria [31]. These anthropogenic disturbances, largely absent in natural forests, render orchards fundamentally distinct in nutrient cycling and carbon sequestration trajectories [28]. Thus, elucidating how management practices interact with stand age to shape C:N:P stoichiometry and carbon stocks is critical for developing sustainable nutrient optimization strategies in apple orchards.

Although previous studies have documented carbon sequestration characteristics [32] and divergent accumulation of particulate and mineral-associated organic C in apple orchards [33], the effects of stand age on C:N:P stoichiometry and associated stocks in apple plantations remain poorly understood. To address this gap, this study examines the ecosystem C:N:P stoichiometry and stocks across a chronosequence of *Malus pumila* orchards in the eastern Yan Mountains, Hebei Province, North China. Specifically, we aim to (1) quantify the C, N, and P concentrations and ecosystem C stocks in different tree tissues (root, stem, branch, and foliage) and soils along stand development stages; (2) analyze variations in C:N:P stoichiometry within the plant–soil system and identify key nutrient limitations for tree growth; and (3) explore coupling relationships between plant and soil nutrients. We hypothesized that (1) stand age would significantly alter C:N:P stoichiometry in plant tissues and soils, with decreasing N and P concentrations and increasing C:N and C:P ratios in tree tissues indicating progressive nutrient limitation, and (2) plant and soil nutrient pools would be weakly coupled in these intensively managed orchards due to anthropogenic disruption. This research will provide critical insights into biogeochemical cycles in apple orchards and offer a scientific basis for sustainable management and nutrient optimization strategies in the region.

## 2. Results

### 2.1. Tree Nutrient Concentrations and Stoichiometric Ratios

**Figure 1:**
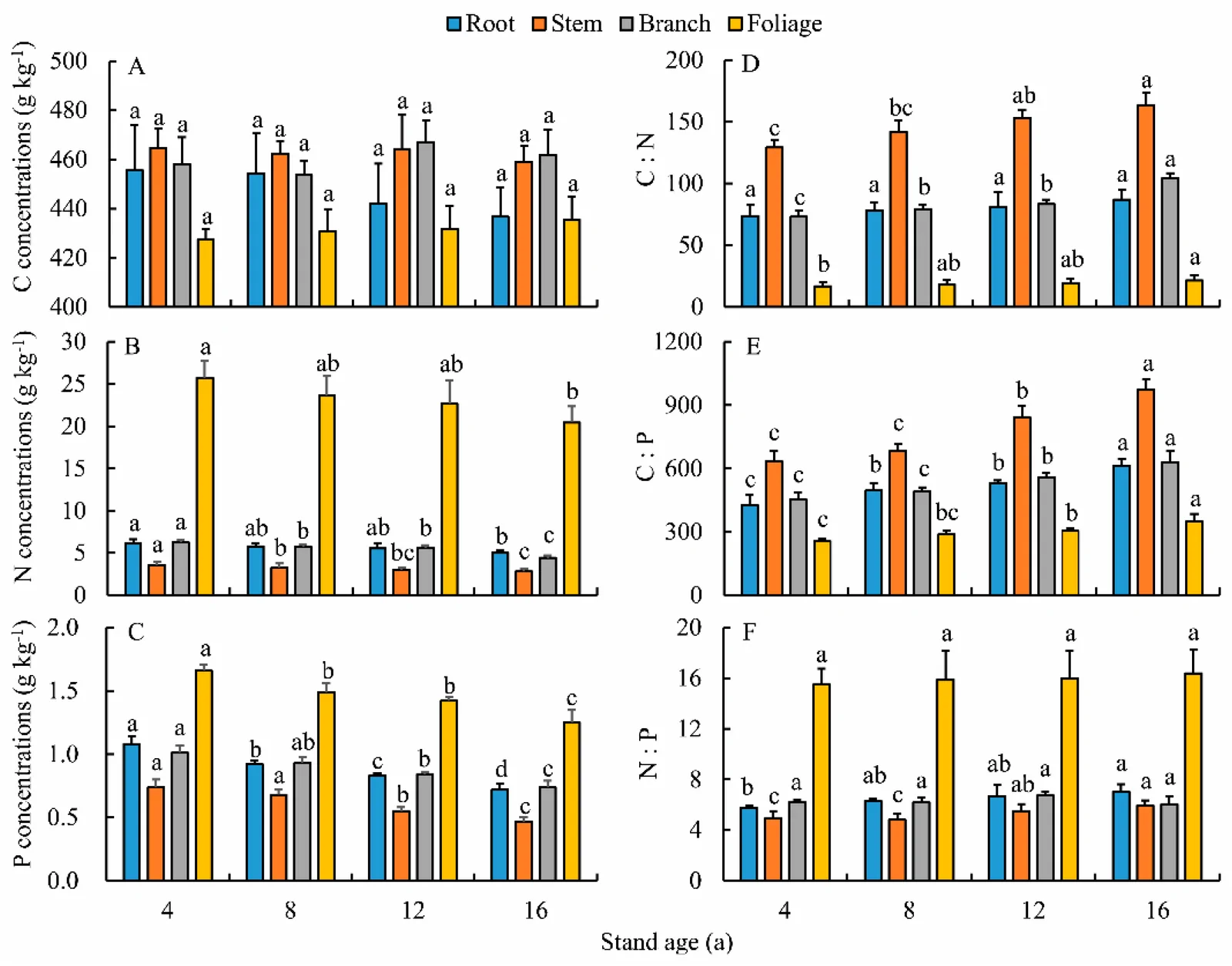
Tree carbon (C), nitrogen (N), phosphorus (P) concentrations and stoichiometric characteristics of *M. pumila* plantations at different stand ages (mean ± S.D.). (**A**) C concentrations; (**B**) N concentrations; (**C**) P concentrations; (**D**) C:N; (**E**) C:P; (**F**) N:P. For each tissue type, different lowercase letters indicate significant differences among stand ages (*p* < 0.05).

The concentrations of C, N, and P varied among root, stem, branch, and foliage tissues of *M. pumila* trees (Figure 1A–C). Across all tissues, the highest C concentration (467.03 g kg^−1^) was observed in branches of the 12 yr stand and the lowest (427.42 g kg^−1^) in foliage of the 4 yr stand (Figure 1A). While C concentrations in roots, stems, and branches showed no age-dependent trend, foliage C concentration increased non-significantly with stand age (*p* > 0.05) (Figure 1A). In contrast, N and P concentrations in all four tissues decreased significantly as stand age increased (Figure 1B,C). Among tissues, foliage had the lowest C concentration but the highest N and P concentrations. With increasing stand age, the C:N and C:P ratios in all tissues, as well as the N:P ratios in roots and stems, rose significantly (*p* < 0.05) (Figure 1D–F). The N:P ratios in branch and foliage also tended to increase along the chronosequence, though the changes were not statistically significant. Across tissues, stems exhibited the highest C:N and C:P ratios, whereas foliage had the highest N:P ratios.

### 2.2. Nutrient Concentrations and Stoichiometric Ratios in Soil

In each soil layer, C, N, and P concentrations first decreased and then increased along the chronosequence, with significant differences among stand ages (*p* < 0.05) (Table 1). The highest mean soil C, N, and P concentrations in the 16 yr stand were 9.51, 0.85, and 0.63 g kg^−1^, respectively. In the deepest layer (60–100 cm), concentrations varied less with age, though the 16 yr stand still exhibited relatively higher values. Vertically, for all stand ages, all three nutrient concentrations decreased with soil depth, peaking in the 0–20 cm layer and reaching their lowest values in the 60–100 cm layer.

Stand age significantly influenced the C:N and C:P ratios across all soil layers, as well as the N:P ratio in the 0–20 cm layer (*p* < 0.05) (Table 1). However, at depths of 20–40, 40–60, and 60–100 cm, the N:P ratio did not differ significantly among the four stand ages. Across ages, the N:P ratio remained relatively stable, ranging mostly from 1.1 to 1.7, with slightly lower surface layer values at the 12 and 16 yr stands (1.3–1.4) compared to the 4 yr stand (1.7). Within each stand age, both the C:N and C:P ratios tended to decline with soil depth, whereas the N:P ratio remained largely unchanged across soil depths, consistently between 1.1 and 1.7.

### 2.3. Relationships Between Plant and Soil Nutrient Concentrations

**Figure 2:**
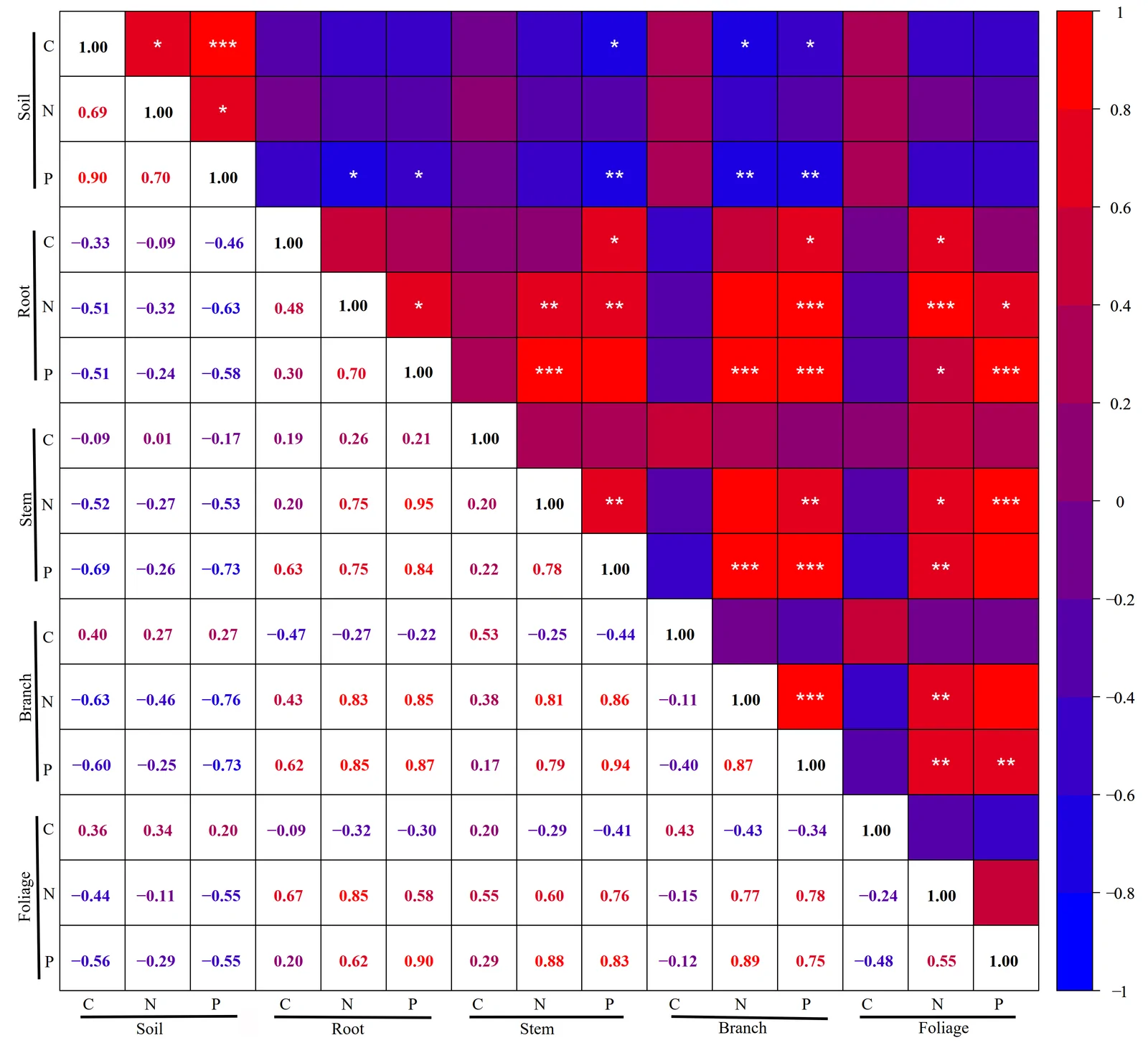
Pearson’s correlations between tree and soil carbon (C), nitrogen (N), and phosphorus (P) concentrations of *M. pumila* plantations. ***, **, and * represent *p* < 0.001, *p* < 0.01, and *p* < 0.05, respectively. The font colors indicate the direction of correlation: red for positive correlations and blue for negative correlations.

Soil C, N, and P concentrations were all significantly positively intercorrelated (Figure 2). Across different tree tissues, root N showed significant positive correlations with root P, stem P, branch P, and foliage P (*p* < 0.05 or *p* < 0.01). Similarly, root P was significantly positively correlated with N and P in stem, branch and foliage tissues (*p* < 0.01 or *p* < 0.05). Stem N and P, as well as branch N and P, were all significantly positively correlated with each other. In contrast, C concentrations in stem, branch, and foliage tissues generally showed few significant correlations with any other tree nutrients. In the soil–tree system, most correlations between soil nutrients and plant nutrient concentrations were negative. Specifically, soil P showed consistent negative correlations with root N, root P, stem P, branch N, and branch P (*p* < 0.01 or *p* < 0.05). Soil C was negatively correlated with stem P, branch N, and branch P (*p* < 0.05). No significant correlations were found between soil nutrients and C concentrations in any plant tissue (*p* > 0.05). Taken together, these predominantly negative and largely non-significant relationships underscore the weak plant–soil nutrient coupling characteristic of this intensively managed orchard ecosystem.

### 2.4. Ecosystem C Stocks Across Different Stand Age Sequences

The total ecosystem C stocks across the four stand ages ranged from 70.80 to 136.13 Mg ha^−1^ (Table 2). Along the chronosequence, the total ecosystem C stocks first decreased from 83.51 Mg ha^−1^ (4 yr) to 70.80 Mg ha^−1^ (8 yr) and then increased progressively to 102.00 Mg ha^−1^ (12 yr) and 136.13 Mg ha^−1^ (16 yr), with significant differences among stand ages (*p* < 0.05). The C stocks of all tree tissues (root, stem, branch, and foliage) increased significantly with stand age (*p* < 0.05). The soil C stocks also showed an initial decline, followed by a subsequent increase along the chronosequence. Within the 0–60 cm soil layer, the C stocks decreased with increasing depth at each stand age. In this study, the ecosystem C stock was predominantly contributed by the soil layer, accounting for 84.7% to 99.7% of the total, whereas the tree layer contributed only 0.3% to 15.3%.

## 3. Discussion

### 3.1. Effects of Stand Age on CNP Concentrations and Stoichiometries in Plants

Stand age is an important factor influencing the changes in tree layer C concentrations, with different tissues exhibiting varying trends along the chronosequence [34,35]. In this study, foliage C concentration increased with stand age (Figure 1A). In contrast, no clear age-related patterns in C concentrations of root, stem, and branch were observed, which is consistent with findings in *Pinus koraiensis* plantations of central Korea [36] and in *Metasequoia glyptostroboides* forests in coastal China [35]. However, other studies have reported that C concentrations in all tree tissues increased with stand age in *P. massoniana* forests in the Sichuan Province [37] and *Acacia mangium* plantations in southeastern Vietnam [38]. In addition, C concentrations in various tissues of *Betula platyphylla* forests in the cold-temperate zone of China decreased significantly with stand age [39]. In the present study, the range of C concentrations in tree tissues was 427.42–467.03 g kg^−1^, similar to that in *R. pseudoacacia* on the Loess Plateau (380.9–470.4 g kg^−1^) [40] and in *Sonneratia apetala* mangroves in southern China (367–496 g kg^−1^) [41], but lower than those in *P. massoniana* in the Sichuan Province (450–555 g kg^−1^) [37]. This indicates that tree layer C concentrations are also influenced by vegetation type [20]. Moreover, even for the same tree species, C concentrations may vary due to differences in climatic conditions and site characteristics [42,43].

N and P are two essential nutrients that are often in short supply in plants, and they are important factors limiting the productivity of terrestrial ecosystems [44,45]. At different stand ages, the photosynthetic capacity and nutrient demand of plants vary greatly, which directly leads to different trends in N and P concentrations among tissues [46,47]. Zhang et al. [35] found no clear age-related pattern in N and P concentrations in various tissues of *M. glyptostroboides* plantations in the eastern Jiangsu Province. Liu et al. [40] reported that N concentrations in the foliage and root of *R. pseudoacacia* forests on the Loess Plateau increased with stand age. In the present study, N and P concentrations in all tissues of *M. pumila* trees decreased significantly with stand age (Figure 1B,C). The possible reasons include: (1) a gradual decline in root N and P uptake, branch N and P transport and stocks, and leaf N and P metabolism, resulting in reduced N and P concentrations in all tissues [48] and (2) the age-related increase in woody biomass, which dilutes N and P concentrations in various tissues [35]. In addition to stand age, N and P concentrations are also influenced by factors such as tree species composition, altitude, and climatic conditions [20,49]. For example, N and P concentrations in various tissues of the N-fixing tree species *R. pseudoacacia* on the Loess Plateau were significantly higher than those in the non-N-fixing species *P. tabuliformis* [50]. In the woody plants of eastern Chinese forests, stem N and P concentrations decreased with increasing altitude [51]. At the community level, stem N and P concentrations of terrestrial plants in China showed a decreasing trend with increasing mean annual temperature/precipitation [45]. In this study, the mean N and P concentrations in foliage across different stand ages (23.12 and 1.46 g kg^−1^, respectively) were significantly higher than those in other tissues, and they fell within the ranges reported for plant foliage in China’s terrestrial ecosystems [52] and globally [53].

Compared with other tissues in the tree layer, the stem has a higher proportion of C-rich compounds such as lignin and therefore exhibits higher C:N and C:P ratios [13,44]. The results of the present study also confirmed this pattern (Figure 1D,E). The C:N ratio in all tissues of *M. pumila* trees increased significantly with stand age, which is consistent with the findings of Yang and Luo [54]. In contrast, in other N-fixing tree species, tree growth requires more N-rich substances (such as enzymes, transport proteins, and amino acids) to participate in metabolic activities (e.g., photosynthesis and respiration), leading to increased N concentrations in various tissues and consequently a decreasing trend in C:N with stand age [9,12]. In this study, C concentrations in *M. pumila* trees did not change significantly with stand age, whereas P concentrations decreased significantly, resulting in a significant increase in C:P ratios. This is consistent with Zhang et al. [35], who reported that tree C:P increased along the chronosequence of *M. glyptostroboides*. The foliage C:N and C:P ratios indicate the capacity of plants to assimilate C relative to nutrient uptake and to some extent reflect nutrient use efficiency and plant growth rate [55]. As shown in Figure 1D,F, the 4 yr *M. pumila* stand had the lowest foliage C:N and C:P ratios, suggesting the lowest nutrient use efficiency.

The foliage N:P ratio is a useful indicator of N or P limitation [56]. Generally, N:P < 14 indicates N limitation, 14–16 suggests co-limitation by N and P, and >16 points to P limitation [57]. In this study, the foliage N:P ratios of 4 and 8 yr *M. pumila* stands were 15.49 and 15.89, respectively, indicating co-limitation by N and P in the soil. At older ages, the ratios exceeded 16, suggesting a shift to P limitation. This trend of increasing N:P ratios from below 14 to above 16 along the chronosequence has also been observed in *Larix kaempferi* and *M. glyptostroboides* plantations [35,47]. These findings imply that plantation growth transitions from relative N limitation to relative P limitation with stand age, reflecting the combined influence of biotic factors (e.g., species and functional groups) and abiotic factors (e.g., temperature, moisture, light, and soil nutrients) on foliage N:P ratios [58].

The progressive intensification of P limitation with increasing stand age in *M. pumila* orchards observed in this study can be attributed to several interacting mechanisms. First, as apple trees mature, biomass accumulation accelerates substantially, leading to greater P demand for metabolic processes such as photosynthesis, respiration, and nucleic acid synthesis, while soil P supply becomes increasingly insufficient to meet this growing demand [59]. Second, long-term continuous cultivation without adequate P replenishment gradually depletes soil available P pools, particularly in the surface layer where root activity is concentrated [60]. Third, the continuous application of N fertilizers, a common practice in apple orchards, may exacerbate the shift from N to P limitation. This practice stimulates vegetative growth and further increases P demand without corresponding P inputs, thereby aggravating the stoichiometric imbalance between N and P [61]. Fourth, soil enzyme activities and microbial metabolic processes also play a critical role in modulating nutrient limitation [62]. Collectively, these biotic (increasing biomass P demand, age-dependent microbial dynamics) and abiotic (soil P depletion, N-induced stoichiometric imbalance, physical constraints on P diffusion) factors converge to intensify P limitation as orchards age, highlighting the need for targeted P management strategies in mature apple production systems [26,62].

### 3.2. Effects of Stand Age on CNP Concentrations and Stoichiometries in Soils

Soil depth critically modulates microbial diversity and nutrient cycling, with functional genes associated with C, N, and P cycles exhibiting pronounced vertical stratification and greater biogeochemical activity in surface layers [63]. Along the stand chronosequence, increasing exogenous inputs from litter and root decomposition [64], coupled with improved soil biological activity (microbial biomass and enzyme activity) driven by changes in light, moisture, pH, and temperature [9,65], collectively enhance soil C, N, and P concentrations. These depth-dependent and age-related patterns together regulate soil C:N:P stoichiometry and ecosystem functioning [66,67,68].

In this study, the contents of C, N, and P in each soil layer of *M. pumila* orchards among different ages showed significant differences, with a trend of first decreasing and then increasing along the chronosequence (Table 1). This trend is largely attributable to the interactive effects of stand development and anthropogenic management practices. In the early stage (4 yr orchard), intercropping with food crops (e.g., corn) and intensive tillage and fertilization practices are common in this region, as apple trees have not yet entered the fruit-bearing period. These practices significantly enhance soil C, N, and P inputs through crop residue incorporation, organic matter addition, and fertilizer application [27]. Meta-analyses have confirmed that intercropping systems can increase soil organic matter content by up to 40% in the surface layer, while tillage and fertilization practices significantly improve soil nutrient availability [69]. After entering the fruit-bearing period (8 yr orchard), canopy closure typically leads to the cessation of intercropping, and the reduction in exogenous organic matter inputs combined with continued nutrient removal through fruit harvesting result in a decline in soil C, N, and P concentrations. With further stand development (12 and 16 yr orchards), the accumulation of litter decomposition and root growth and metabolism enrich soil C, N, and P concentrations. These results are consistent with the well-established view that soil C, N, and P usually increase with forest age or secondary forest succession [70,71]. Moreover, ground cover management practices, such as natural grassing, have been shown to significantly increase soil C (20.0%), N (15.0%), and P (13.0%) concentrations in orchards [72].

It is worth noting that anthropogenic management not only directly adds nutrients but also indirectly modulates soil C:N:P stoichiometry by altering microbial community composition and enzyme activities [30]. Thus, the U-shaped trajectory of soil nutrients along the chronosequence reflects the dynamic balance between anthropogenic nutrient inputs (fertilization, intercropping, ground cover management) and outputs (fruit harvesting, nutrient leaching), superimposed on natural processes of litter accumulation and decomposition. This finding underscores that sustainable orchard management must carefully balance short-term productivity gains with long-term soil fertility maintenance. Furthermore, the C, N, and P contents in each soil layer of *M. pumila* orchards of different ages decreased with increasing soil depth, a pattern also observed in studies of tropical forests [68] and subtropical forests [67]. This trend could be attributed to the decreasing contribution of humus and soil organic matter derived from above- and belowground litter with increasing soil depth [73].

The soil C:N:P stoichiometries are key indicators reflecting soil nutrient cycles and ecosystem functioning in different vegetation types [73,74]. In this study, the trends in C:N, C:P, and N:P across soil layers of *M. pumila* orchards of different ages were generally consistent with those of soil C, N, and P concentrations, showing a pattern of first decreasing and then increasing. The C:N ratios in the soil layers of *M. pumila* orchards of different ages ranged from 7.4 to 12.3, falling within the range reported for other plantations [15,75]. The mean C:P and N:P ratios in the soil layers of *M. pumila* orchards were 13.8 and 1.4, respectively, which are substantially lower than those reported for other plantations [35,67]. This is mainly because *M. pumila* orchards are greatly influenced by anthropogenic management practices such as fertilization and tillage during growth, resulting in higher soil N and P concentrations and consequently leading to lower C:P and N:P ratios.

### 3.3. Correlations Among C, N, and P in Plant and Soil

Soil and plants have complex interactions, as they have developed together over long periods [12,18]. In this study, correlation analysis revealed that soil P concentration in the *M. pumila* orchards was significantly negatively correlated with root N and P concentrations, stem P concentration, and branch N and P concentrations (*p* < 0.01 or *p* < 0.05) (Figure 2). Under anthropogenic management practices for *M. pumila* orchards, these results could be attributed to the following three mechanisms. First, high soil P boosts biomass growth, diluting N and P concentrations in tissues [76,77]. Second, trees reduce P uptake and mycorrhizal activity when soil P is high, also limiting N absorption [78]. Third, high soil P increases fruit yield, and harvested fruits remove large amounts of N and P [79]. In contrast, no significant correlations have been found between soil and foliage in *M. pumila* orchards. A previous study also reported that soil nutrients were weakly correlated with foliage nutrients in Scots pine (*Pinus sylvestris*) and Norway spruce (*Piea abies*) forests in Sweden [80]. These results suggest that coupling relations of major nutrients between soils and foliage did not always exist [13].

In plant biomass, root N and P levels were significantly and positively correlated with those in aboveground tissues (i.e., stem, branch, and foliage) (Figure 2), aligning with observations from previous studies [45,50]. Such cross-organ relationships point to a coordinated pattern of nutrient allocation between belowground and aboveground parts [81]. Specifically, plants tend to maintain consistent N status across different organs while also coupling root N with aboveground P accumulation. This linkage likely mirrors the tight functional integration of roots and aboveground tissues in nutrient uptake and photosynthetic metabolism [53].

Significant positive correlations among soil C, N, and P concentrations were observed in this study (Figure 2), indicating a strong coupling of these key nutrients in the *M. pumila* orchard soil system. This coupling is commonly driven by shared biogeochemical processes, such as the simultaneous accumulation and mineralization of organic matter [82,83]. The consistency of our findings with previous studies conducted on the Loess Plateau, China [9,18], further supports the view that long-term soil development under similar climatic and geomorphic conditions leads to synchronized nutrient dynamics. Our results not only reinforce the generality of this pattern across different sites but also suggest that management practices aimed at increasing soil organic matter (e.g., fertilization and cultivation) are likely to enhance N and P availability simultaneously, which has important implications for soil fertility and ecosystem productivity [84,85].

### 3.4. C Stocks

The chronosequence approach is commonly employed to simulate stand development dynamics [86] and allows the study of forest growth processes for different tree species [41,73,87]. Consistent with Chen et al. [88] and Justine et al. [37], tree biomass C stocks in *M. pumila* orchards showed an increasing trend with stand age, as C accumulation in tree biomass is strongly correlated with an increase in wood biomass. In this study, due to anthropogenic management practices, the soil C stocks of *M. pumila* orchards showed a trend of first decreasing and then increasing with stand age (Table 2). Similar trends were also reported by Deng et al. [89] and Wang et al. [71], who found a decrease in soil C stock at an early age, which then gradually increased with age. Under natural conditions, the soil C stocks of plantations such as *Betula pendula* [86], *Zanthoxylum bungeanum* [90], and *P. massoniana* [37] all showed an increasing trend with stand age. However, Mao et al. [14] determined that the amount of soil C stocks firstly increased and then decreased with increasing stand age. These discrepancies may be partly due to other influencing factors (e.g., climate conditions, soil characteristics, species type, management practices, litter input, root metabolism, and previous land use) overshadowing the effect of stand age [43,91,92]. Moreover, fallow duration and land use history also critically influence soil C and N dynamics. Longer fallow periods have been shown to enhance vertical movement and stabilization of soil C and N, with enriched δ^13^C and δ^15^N at depth indicating microbial transformation and fire legacy effects [93,94]. Our observed recovery of soil C stocks in 12 and 16 yr orchards aligns with these age-dependent patterns, suggesting that prolonged stand development promotes both surface C accumulation and its stabilization in deeper soil horizons.

The total mean ecosystem C stock of 98.11 Mg ha^−1^ observed here is considerably higher than the value of 40.0 Mg ha^−1^ reported as the average vegetation carbon stock in China [95], but much lower than the average carbon stock of global forest ecosystems (163.1 Mg ha^−1^) [96]. The carbon stocks in the soil layer of the *M. pumila* orchards accounted for 84.7–99.7% of the total ecosystem carbon stocks, which is higher than the contributions of the soil layer in 30 yr pure and mixed forests in subtropical China (63.5–84.7%) [97] and in 40 yr plantations and secondary forests on the Loess Plateau (58.0–75.1%) [50]. This is mainly because the *M. pumila* orchards in this study are relatively young, with relatively low carbon stocks in the tree layer, which accounts for a small proportion of the total ecosystem C stocks.

## 4. Materials and Methods

### 4.1. Site Description

**Figure 3:**
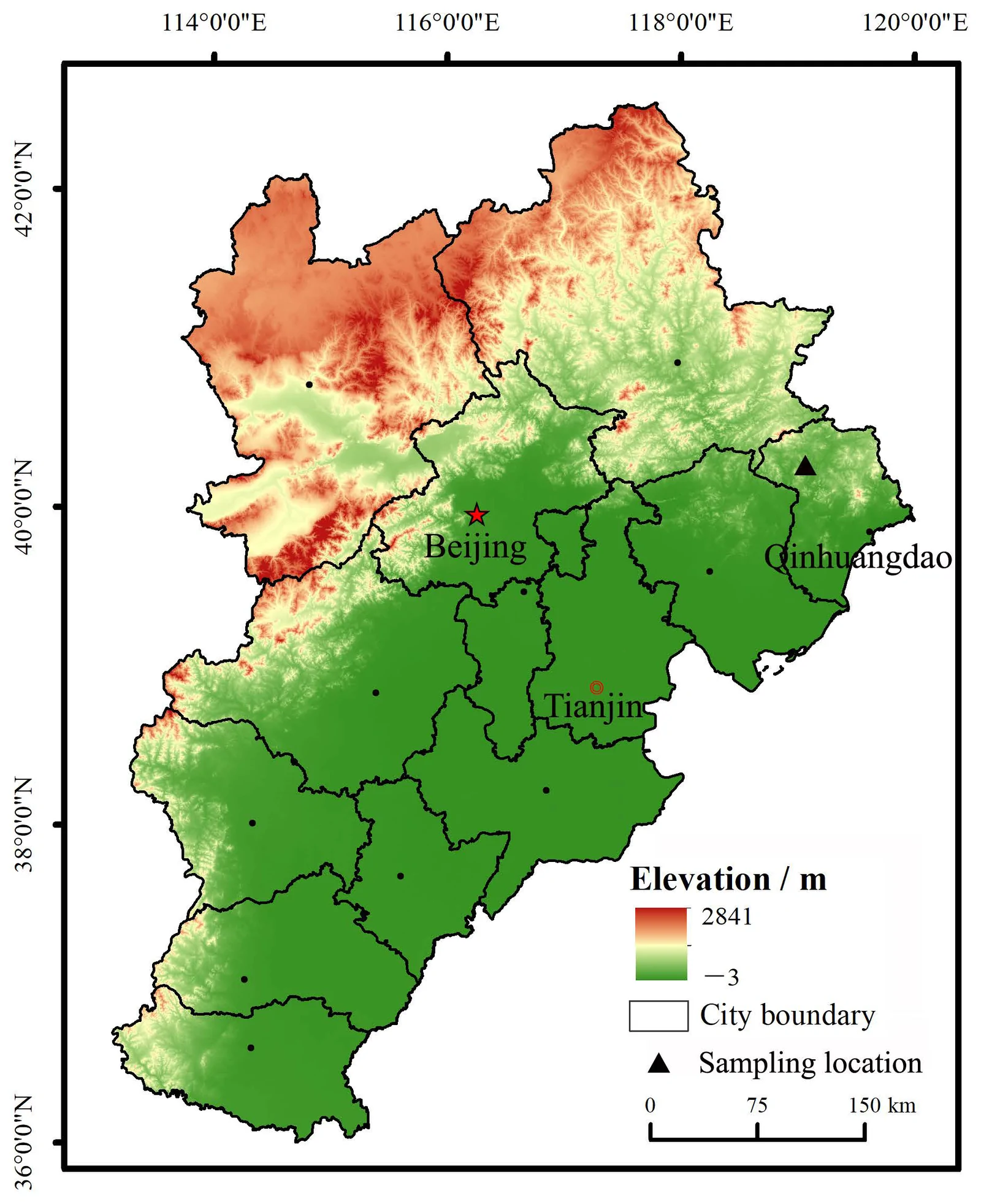
Geographical location of the study site in Hebei Province, North China.

This study was carried out in the Qinglong Manchu Autonomous County (40°04′–40°36′ N, 118°33′–119°36′ E), situated northwest of Qinhuangdao City in the northern Hebei Province, China (Figure 3). The area features a temperate sub-humid continental monsoon climate, with an average elevation of roughly 250 m above sea level. The mean annual temperature is approximately 8.9 °C, reaching a maximum of 29.7 °C in July and a minimum of −13.6 °C in January. The mean annual precipitation is around 715 mm, with most rainfall occurring from July to August. The local soils are classified as Cinnamon soil [98], which corresponds to Haplustalf under the U.S. soil taxonomy system [99].

### 4.2. Field Sampling and Measurements

In August and September 2020, *M. pumila* orchards with similar site conditions (e.g., altitude, slope position, slope direction, and soil type) were selected and classified into four age groups (4, 8, 12, and 16 yr). For each age group, three independent plots of 20 m × 20 m were established. Within each plot, tree height, crown width, and basal diameter (measured at 10 cm above ground) were recorded. The stand characteristics are summarized in Table 3. Based on the plot survey, a total of 12 trees (3 trees per age group × 4 age groups) were selected for destructively harvesting to estimate biomass.

The sample trees were felled using a chainsaw at the ground level [100]. Branches were removed from each main stem, and both stems and the branches (together with foliage) were weighed in the field [87]. Based on branch diameter classification, five leafy branch samples were randomly selected to estimate the biomass proportions of branches and foliage. The sampled branches and foliage were separated and weighed individually. The resulting leaf-to-branch mass ratio was subsequently applied to compute the whole-tree fresh biomass of foliage and branches [101]. Coarse roots (diameter > 5 mm) were carefully excavated through a combination of manual digging and mechanical traction and then collected for biomass determination [102].

For each tree component, a 100 g subsample was taken to determine the fresh-to-dry mass ratio at 80 °C until constant weight. These ratios were then used to convert the fresh mass of all tree components into dry biomass [87,103]. The tree height of the M. pumila plantations was affected by anthropogenic disturbances such as pruning during growth. Therefore, allometric regression equations between biomass and basal diameter were developed across this chronosequence. These allometric equations were well fitted for each tree component (*R*^2^ > 0.9, *p* < 0.01) (Table 4). Based on these equations, the biomass of each tree was calculated, and the total biomass for each stand was further determined [88]. In addition, owing to management practices such as plowing and weeding, the herb and litter layers within the plantations were sparse. Consequently, no sampling was conducted for these two layers.

Soils were sampled to the depth of 100 cm. Within each plot, five sampling points were systematically arranged following Chen et al. [88]. At each point, three subsamples were collected from four depth intervals: 0–20, 20–40, 40–60, and 60–100 cm. These subsamples were then combined by sampling point and depth layer, as described by Wu et al. [58] and Xu et al. [104]. All composite samples were transported to the laboratory and air-dried for nutrient concentration analysis. Meanwhile, undisturbed soil cores (100 cm^3^ in volume) were extracted to determine bulk density [50].

### 4.3. Nutrients Analysis and Stocks Calculation

The dried plants and soil samples were mechanically ground and passed through a 0.25 mm sieve for nutrient analysis [90]. Organic C concentrations in the plant and soil samples were determined using the H_2_SO_4_–K_2_Cr_2_O_7_ oxidation method [41]. For the total N and P, the plant and soil samples were digested with H_2_SO_4_–H_2_O_2_, after which N was measured by the micro-Kjeldahl method and P by the colorimetric method using an UV spectrophotometer, respectively [40].

The total plant C stocks were summed for all tree components. The C stocks of different tree components were calculated with Equation (1) [67]:

(1) Tree stock (Mg ha^−1^) = tree component biomass (Mg ha^−1^) × tree C concentrations

The total soil C stocks within the 100 cm layer were summed for all four soil layers. To estimate the soil C stocks in each soil layer, the following Equation (2) [105] was used:

(2) Soil stock (Mg ha^−1^) = SOC concentrations (g kg^−1^) × bulk density (g cm^−3^) × layer thickness (cm) × (1 − *α*) × 0.1

where *α* is the volume fraction of gravel (%).

### 4.4. Data Analysis

C, N, and P concentrations in tree components and soil were measured as the mass concentrations, and their stoichiometric ratios were expressed as a mass ratio. Data processing and figures were prepared using Microsoft Excel 2016 software (Microsoft Inc., Redmond, WA, USA). One-way analysis of variance (ANOVA) with Duncan’s post hoc test (*p* < 0.05) was applied to assess differences in nutrient concentrations, stoichiometries, and the C stocks across stand ages [88]. The results are presented as mean ± standard deviation (S.D.). All statistical analyses were performed with R software (version 4.5.2, Hornik and R Core Team, Vienna, Austria). The Pearson correlation analysis between C, N, and P concentrations in tree components and soil was conducted using the “corrplot” package [106] in R.

## 5. Conclusions

This study presents a comprehensive assessment of C:N:P stoichiometric dynamics and ecosystem carbon stocks across a chronosequence of apple orchards in North China, revealing that stand age and anthropogenic management jointly shape the stoichiometric balance and carbon sequestration trajectory of these perennial agroecosystems. Our results demonstrate a progressive shift from N–P co-limitation to P limitation with increasing stand age, driven by increasing biomass P demand, continuous N fertilization without proportional P replenishment, and age-dependent microbial dynamics. Soil C, N, and P concentrations followed a U-shaped trajectory along the chronosequence, reflecting a dynamic balance between anthropogenic nutrient inputs (intercropping, fertilization, ground cover management) and outputs (fruit harvesting, nutrient leaching). The predominantly negative and largely non-significant correlations between soil and plant nutrient pools indicate weak plant–soil nutrient coupling in these intensively managed orchards, a feature that distinguishes them from natural forest ecosystems.

From a management perspective, alleviating P limitation in mature orchards (≥12 yr) through targeted P fertilization or enhanced P mobilization via cover cropping and microbial amendments is essential for sustaining productivity. Given that soil accounts for 84.7–99.7% of the total ecosystem C stocks, maintaining soil organic matter inputs through organic manure application, retention of ground cover vegetation, and reduced tillage is critical not only for soil fertility but also for long-term carbon sequestration. The weak plant–soil nutrient coupling further suggests that soil nutrient status alone is an unreliable predictor of tree nutrient demand; integrated nutrient management should therefore combine soil testing with plant-based diagnostic tools (e.g., leaf nutrient analysis) to optimize fertilization regimes. Collectively, these findings underscore that sustainable management of apple orchards in North China must simultaneously address P limitation in mature stands, maintain soil organic matter to preserve the large soil C pool, and adopt integrated nutrient diagnosis to reconcile tree growth, fruit yield, and long-term soil fertility under continuous cultivation.

## Figures and Tables

**Table 1:** Soil carbon (C), nitrogen (N), phosphorus (P) concentrations and stoichiometric characteristics of *Malus pumila* orchards in different ages in Northern China (mean ± S.D.).

Stand Age (a)	Soil Depth (cm)	C (g kg^−1^)	N (g kg^−1^)	P (g kg^−1^)	C:N	C:P	N:P
4	0–20	11.51 ± 0.20 b	1.13 ± 0.02 a	0.66 ± 0.01 c	10.2 ± 0.2 b	17.5 ± 0.6 a	1.7 ± 0.1 a
	20–40	8.23 ± 1.18 b	0.78 ± 0.04 b	0.57 ± 0.01 b	10.7 ± 2.1 a	14.5 ± 2.3 ab	1.4 ± 0.1 a
	40–60	6.66 ± 0.19 b	0.69 ± 0.01 bc	0.51 ± 0.02 ab	9.6 ± 0.3 ab	13.2 ± 0.2 b	1.4 ± 0.1 a
	60–100	4.58 ± 0.62 bc	0.62 ± 0.01 a	0.42 ± 0.02 b	7.4 ± 1.0 b	10.1 ± 1.0 bc	1.4 ± 0.1 a
8	0–20	8.35 ± 0.36 c	0.93 ± 0.16 b	0.57 ± 0.01 d	9.2 ± 1.1 b	14.7 ± 0.9 c	1.6 ± 0.3 ab
	20–40	6.23 ± 0.73 c	0.69 ± 0.05 c	0.52 ± 0.02 c	9.1 ± 1.8 b	12.0 ± 1.3 c	1.3 ± 0.1 a
	40–60	5.03 ± 0.15 c	0.61 ± 0.07 c	0.47 ± 0.03 c	8.3 ± 0.9 b	10.7 ± 0.8 c	1.3 ± 0.2 a
	60–100	4.04 ± 0.27 c	0.54 ± 0.07 b	0.38 ± 0.02 b	7.5 ± 0.6 b	9.5 ± 0.9 c	1.3 ± 0.2 a
12	0–20	11.59 ± 0.11 b	0.94 ± 0.05 b	0.73 ± 0.03 b	12.3 ± 0.6 a	15.9 ± 0.2 b	1.3 ± 0.1 c
	20–40	8.76 ± 0.51 ab	0.76 ± 0.01 bc	0.52 ± 0.02 c	11.6 ± 0.6 a	16.9 ± 0.4 a	1.5 ± 0.1 a
	40–60	7.50 ± 0.29 a	0.67 ± 0.04 c	0.48 ± 0.01 c	11.3 ± 0.7 a	15.6 ± 0.2 a	1.4 ± 0.1 a
	60–100	5.26 ± 0.39 b	0.53 ± 0.01 b	0.46 ± 0.01 ab	10.0 ± 0.8 a	11.3 ± 0.8 b	1.1 ± 0.1 a
16	0–20	13.82 ± 0.12 a	1.14 ± 0.05 a	0.84 ± 0.02 a	12.1 ± 0.5 a	16.4 ± 0.5 ab	1.4 ± 0.1 bc
	20–40	9.89 ± 0.61 a	0.86 ± 0.04 a	0.65 ± 0.03 a	11.6 ± 1.2 a	15.2 ± 1.6 a	1.3 ± 0.1 a
	40–60	7.72 ± 0.64 a	0.76 ± 0.02 a	0.54 ± 0.01 a	10.2 ± 1.2 a	14.4 ± 0.9 b	1.4 ± 0.1 a
	60–100	6.59 ± 0.45 a	0.65 ± 0.03 a	0.49 ± 0.01 a	10.2 ± 1.1 a	13.3 ± 1.1 a	1.3 ± 0.1 a

For each soil depth, different lowercase letters indicate significant differences among stand ages (*p* < 0.05).

**Table 2:** Carbon (C) stocks in various ecosystem components of *M. pumila* orchards at different stand ages (mean ± S.D., Mg ha^−1^).

Stand Age (a)	Tree Layer (Mg ha^−1^)				Soil Layer (Mg ha^−1^)				Ecosystem C Stocks (Mg ha^−1^)
	Root	Stem	Branch	Foliage	0–20 cm	20–40 cm	40–60 cm	60–100 cm	
4	0.08 ± 0.02 d	0.08 ± 0.02 d	0.04 ± 0.01 d	0.03 ± 0.01 c	25.82 ± 1.57 b	19.29 ± 2.07 b	15.78 ± 1.04 c	22.39 ± 3.36 c	83.51 ± 4.82 c
8	0.54 ± 0.11 c	0.58 ± 0.07 c	0.65 ± 0.17 c	0.09 ± 0.04 c	19.49 ± 1.55 c	15.61 ± 2.02 c	12.69 ± 0.93 d	21.15 ± 1.14 c	70.80 ± 3.35 d
12	2.51 ± 0.07 b	2.96 ± 0.19 b	2.42 ± 0.24 b	0.34 ± 0.10 b	25.37 ± 0.94 b	21.37 ± 1.33 ab	19.52 ± 0.48 b	27.51 ± 2.28 b	102.00 ± 4.77 b
16	9.91 ± 0.39 a	5.86 ± 0.24 a	4.54 ± 0.18 a	0.45 ± 0.12 a	31.16 ± 0.94 a	24.72 ± 1.80 a	21.83 ± 1.75 a	37.66 ± 2.28 a	136.13 ± 3.21 a

For each component, different lowercase letters indicate significant differences among stand ages (*p* < 0.05).

**Table 3:** Stand characteristics of *Malus pumila* orchards in different ages in Northern China (mean ± S.D.).

Parameters	Stand Age (a)			
	4	8	12	16
Longitude (°)	119.215	119.218	119.216	119.217
Latitude (°)	40.509	40.507	40.508	40.508
Elevation (m)	280	293	288	251
Basal diameter (cm)	2.67 ± 0.29	6.83 ± 1.26	13.83 ± 1.04	18.33 ± 1.53
Height (m)	2.05 ± 0.13	3.33 ± 0.15	4.03 ± 0.17	5.51 ± 0.50
Density (trees·ha^−1^)	1108 ± 38	950 ± 66	925 ± 50	766 ± 52
Biomass (kg·m^−2^)	0.05 ± 0.01	0.42 ± 0.18	2.24 ± 0.33	3.72 ± 0.56
Fertilization and field management regime	Well-rotted farmyard manure at 30–45 t ha^−1^ yr^−1^ in autumn. Intercropped with corn between rows. No fruit harvesting.	Well-rotted farmyard manure at 30–45 t ha^−1^ yr^−1^ in autumn. Regular pruning in winter and summer. Natural grass, mowed 2–3 times during growing season. Fruit harvesting removes substantial N, P, K.	Well-rotted farmyard manure at 45–60 t ha^−1^ yr^−1^ in autumn. Regular pruning in winter and summer. Natural grass, mowed 2–3 times during growing season. Fruit harvesting removes substantial N, P, K.	Well-rotted farmyard manure at 45–60 t ha^−1^ yr^−1^ in autumn. Regular pruning in winter and summer. Natural grass, mowed 2–3 times during growing season. Fruit harvesting removes substantial N, P, K.

**Table 4:** Biomass equations for the tree components across the *M. pumila* plantation chronosequence.

Tree Component	Allometric Equation	*R* ^2^	*p*
Root	*W* = 0.012*D*^2.527^	0.883	<0.01
Stem	*W* = 0.015*D*^2.365^	0.959	<0.01
Branch	*W* = 0.007*D*^2.611^	0.965	<0.01
Foliage	*W* = 0.010*D*^1.686^	0.968	<0.01

*W*, dry mass of different components (kg); *D*, basal diameter (stem diameter measured at 10 cm above ground) (cm).

## Data Availability

The original contributions presented in this study are included in the article. Further inquiries can be directed to the corresponding author.
